# Hyaluronan-induced alterations of the gut microbiome protects mice against *Citrobacter rodentium* infection and intestinal inflammation

**DOI:** 10.1080/19490976.2021.1972757

**Published:** 2021-09-30

**Authors:** Tangyou Mao, Chien-Wen Su, Qiaorong Ji, Chih-Yu Chen, Rongjun Wang, Deepak Vijaya Kumar, Jinggang Lan, Lefei Jiao, Hai Ning Shi

**Affiliations:** a Mucosal Immunology and Biology Research Center, Massachusetts General Hospital and Harvard Medical School, Charlestown, USA; b Department of Gastroenterology, Dongfang Hospital, Beijing University of Chinese Medicine, Beijing, China; c Laboratory for Lipid Medicine and Technology, Department of Medicine, Massachusetts General Hospital and Harvard Medical School, Charlestown, USA; d Genetics and Aging Research Unit, Department of Neurology, Massachusetts General Hospital and Harvard Medical School, Charlestown, USA

**Keywords:** Hyaluronan, gut microbiome, *Citrobacter rodentium*, intestinal inflammation, *Akkermansia muciniphila*

## Abstract

Hyaluronan is a glycosaminoglycan polymer that has been shown to play an important role in homeostasis of the gastrointestinal tract. However, its mechanistic significance in gastrointestinal epithelial barrier elements remain unexplored. Here, our results revealed that hyaluronan treatment resulted in significant changes in the gut microbiota in mice. To demonstrate the functional consequences of hyaluronan-treatment and hyaluronan-induced microbiota alterations, *Citrobacter rodentium-* and DSS-induced colitis models and microbiota transplantation approaches were utilized. We showed that hyaluronan alleviated intestinal inflammation in both pathogen and chemically induced intestinal mucosal damage. The protection in bacterial colitis was associated with enhanced *C. rodentium* clearance and alleviation of pathogen-induced gut dysbiosis. Microbiota transplantation experiments showed that the hyaluronan-altered microbiota is sufficient to confer protection against *C. rodentium* infection. Colonization with *Akkermansia muciniphila*, a commensal bacterium that is greatly enriched by hyaluronan treatment, alleviated *C. rodentium*-induced bacterial colitis in mice. Additionally, *Akkermansia*-induced protection was found to be associated with the induction of goblet cells and the production of mucins and epithelial antimicrobial peptides. Collectively, these results provide novel insights into the regulatory role of hyaluronan in modulating the gut microbiota and immunity in enteric infection and inflammation, with therapeutic potential for gut microbiome-targeted immunotherapy.

## INTRODUCTION

Hyaluronan is a linear glycosaminoglycan polymer composed of a repeating disaccharide of β-glucuronic acid and N-acetyl-glucosamine.^1^ Under normal physiological conditions, hyaluronan is typically secreted by cells as a high molecular weight polymer of up to 10,000 kDa, and is present in synovial fluid and the extracellular matrix of many tissues.^2^ During inflammation, large-sized hyaluronan polymers are broken down into smaller fragments, which is typically associated with the activation of hyaluronidases.^3^ It is now recognized that the biological function of hyaluronan fragments depends on its molecular size. In general, high molecular weight hyaluronan with a molecular size >1000 kDa has been suggested to be involved in tissue homeostasis, immune response, regulating inflammation, wound healing, and anti-proliferative effects. It is used extensively in wide varieties of moisturizers and cosmetic preparations,^4^ in addition to being used as effective ingredient in therapeutics to treat osteoarthritis, ophthalmic disease, and cancer.^5,6^ In contrast, low-molecular size hyaluronan has been shown to trigger marked inflammatory response through activation of macrophages and dendritic cells, accompanied by enhanced expression of proinflammatory factors, as well as inhibition of anti-inflammatory factors and their receptors.^7,8^

However, recent emerging evidence also showed that low molecular weight hyaluronan elicited protective effects to suppress the inflammatory response. A recent study demonstrated that hyaluronan fragments <750 kDa played a protective role in the host response to dextran sulfate sodium (DSS)-induced colitis through the activation of Toll-like receptor 4 and the production of prostaglandin E2 via cyclooxygenase-2.^9^ In addition, hyaluronan (45–90 kDa) protected mice from hepatocellular apoptosis during liver injury and alleviated the process of hepatic inflammation in murine models.^10^ Moreover, a 35-kDa hyaluronan has been shown to increase the expression of a human β-defensin-2 ortholog in the colonic epithelium of mice and induce the expression of ZO-1 during *C. rodentium* infection and DSS-induced colitis.^11,12^ In line with these observations, previous studies showed that oral administration of purified hyaluronan, a natural component of human milk, induced antimicrobial peptide release in intestinal mucosa in mice.^13^ These results suggest that orally administered hyaluronan may directly interact with intestinal epithelial cells and affect the innate immune response to intestinal pathogens, suggesting a potential role for exogenous hyaluronan in the modulation of intestinal barrier function. However, whether and how hyaluronan modulates the complex interface between intestinal epithelium, microbiota, and mucosal immunity in the context of enteropathogen infection is unclear. The potential effects of hyaluronan on gastrointestinal microbial communities and, in particular, the functional consequences of hyaluronan-induced gut microbiota, in the modulation of host susceptibility to bacterial enteropathogen and intestinal inflammation, have not been investigated.

The gastrointestinal tract is a complex ecosystem harboring an enormous community of microorganisms that coexist with the host in a symbiotic relationship and play important roles in maintaining human health.^14^ This dynamic interaction between the microbiota and host is conducive to the maintenance of intestinal homeostasis, nutrient acquisition, energy regulation, and colonization resistance against enteropathogens.^15^ Therefore, targeted regulation of the composition and function of intestinal microbiota has been suggested to play an important role in maintaining the homeostatic innate and adaptive immune responses both systemically and in the gut in preventing bacterial enteropathogen infection. In the current study, we tested the hypothesis that hyaluronan-mediated alterations of the gut microbiome significantly modulated mucosal immunity against bacterial enteropathogens and intestinal inflammation. A better understanding of the role of hyaluronan in the regulation of the complex interactions between host gut microbiota, mucosal immune function, and inflammatory processes will facilitate the development of novel therapeutics for many critical diseases.

## RESULTS

### Hyaluronan alters gut microbiome composition and metabolites in Balb/c mice

**Figure 1.**
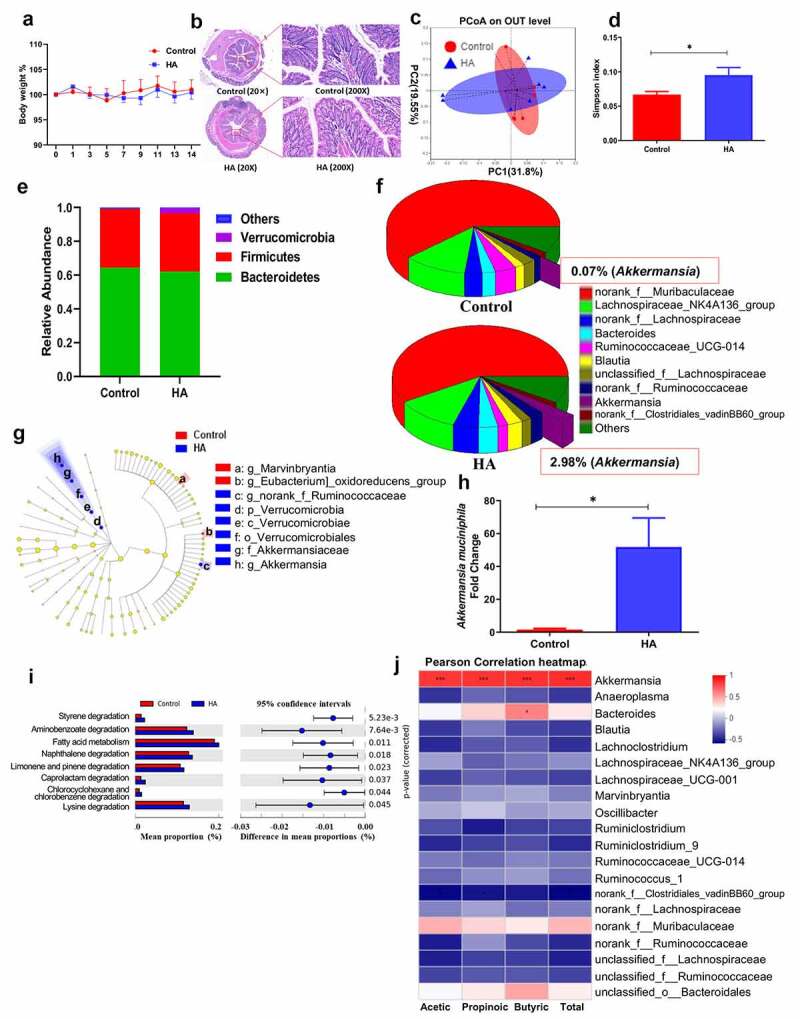
**Hyaluronan treatment selectively alters gut microbiome composition and metabolites in mice**. Balb/c mice were orally gavaged with hyaluronan or PBS once a day for two weeks. **a**. The dynamic change of body weight was monitored daily. **b**. Hematoxylin and eosin staining of the colon sections was conducted after sacrificed and showed no significant effect of hyaluronan on the intestinal morphology. **c**. Principal coordinates analysis (PCoA) analysis showed no difference in gut microbiota structure between the two groups. **d**. Simpson index was assessed and found a remarkable increase in hyaluronan-treated mice. **e, f**. Relative abundance of operational taxonomic units (OTUs) at phylum and genus levels detected by 16s rDNA sequencing in feces of mice fed hyaluronan or PBS. **g**. Cladogram, generated from Linear discriminant analysis Effect Size (LEfSe) analysis, showed differentially abundant taxa of fecal microbiota that were enriched in PBS- and hyaluronan-treated mice. **h**. qPCR analysis revealed a significant increase of *A. muciniphila* in stools from hyaluronan-treated mice. **i**. Microbial community functions was predicted by PICRUSt using STAMP (version 2.1.3). **j**. Pearson correlation of SCFAs and the top 20 genera in each sample. Red represents positive correlation, and blue indicates negative correlation. N = 7 mice in PBS-treated group and 8 mice in hyaluronan-treated mice, ***P < .001, *P < .05 versus the Control group. Significance was determined by a Two-tailed Student’s t-test

To determine whether hyaluronan has effects on healthy BALB/c mice, mice were orally gavaged with hyaluronan or phosphate buffered saline (PBS) once a day for two weeks. Our results showed that hyaluronan treatment had no significant effect on the growth performance and intestinal morphology in mice, suggesting that hyaluronan had no adverse effect on healthy mice (Figure 1a and b). Next, we determined whether hyaluronan inoculation influences the gut microbiota in mice using bacterial 16S rRNA gene sequencing. After removing unqualified sequences, a total of 843,185 raw reads and an average of (60,227.5 ± 2707) reads per sample were obtained. Principal coordinates analysis (PCoA) showed that the gut microbiota structure did not differ significantly between the two groups (Figure 1c). However, a remarkable increase of the Simpson index was detected in hyaluronan-treated mice (Figure 1d), suggesting an improvement in gut microbiota diversity in mice with hyaluronan supplement. In terms of bacterial composition, our results showed that hyaluronan treatment induced significant changes in gut microbiome composition, characterized by a decrease in the abundance of the bacterial genus *Lachnospiraceae_NK4A136_group* and *Ruminococcaceae_UCG-014*, and a marked increase in *Bacteroides* and *Akkermansia*, which belongs to the phylum Verrucomicrobia (Figure 1e, f). Notably, the increase of *Akkermansia* reached approximately 42-fold (0.07% in control mice versus 2.98% in HA-treated mice). Linear discriminant analysis Effect Size (LEfSe) was used for comparison between two groups and biomarker discovery. The results from LEfSe analysis showed that genus *Akkermansia* is the predominant community member and biomarker in hyaluronan-treated mice (Figure 1g). Our qPCR analysis further confirmed that hyaluronan treatment led to a significant increase in *A. muciniphila* (Figure 1h). These results, therefore, demonstrate the role of hyaluronan treatment in promoting the abundance of *Akkermansia*.

In addition to taxonomic composition, the functional profiles of the microbial communities from 16S rRNA gene-based microbial compositions were predicted with the PICRUSt algorithm. Significant differences were detected in 8 KEGG pathways between the two groups (Figure 1i), including the results showing that hyaluronan treatment enhanced activities of fatty acid metabolism (*P* = .011). In the current study, we determined the concentrations of microbiota-derived short-chain fatty acids (SCFAs) in stool by gas chromatography and observed increased levels of acetic acid, butyrate acid, and especially propionic acid in stool of hyaluronan-treated mice. *A. muciniphila* is known to be capable of producing both propionic and acetic acid.^16,17^ Pearson correlation analyses demonstrated a strong positive correlation between SCFAs and the population densities of the genera *Akkermansia* (Figure 1k). These results, therefore, provide strong evidence to demonstrate that hyaluronan feeding induces significant changes in the gut microbiota composition, particularly resulting in enriched abundance of *A. muciniphila* correlated with enhanced production of SCFAs in BALB/c mice. As gut microbiota play crucial roles in host physiology, such as immune modulation, we speculate that hyaluronan-induced alteration in gut microbiota may play a role in the modulation of mucosal inflammatory and immune responses against bacterial enteropathogens.

### *Hyaluronan protects mice against* C. rodentium *infection and intestinal inflammation*

**Figure 2.**
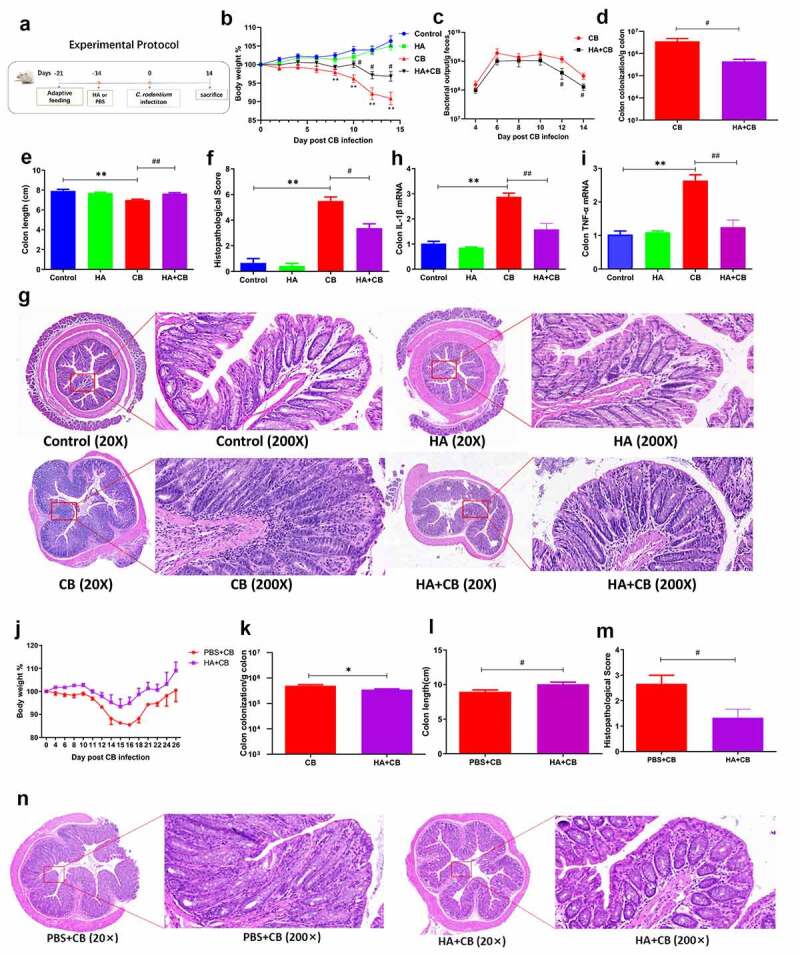
**Pretreatment with hyaluronan protects mice from *C. rodentium* infection**. BALB/c mice were randomly divided into two groups. Mice in HA group were given hyaluronan once a day during the experimental period, and mice in Control group were treated the same volume of PBS. After two weeks of intervention, sub-group of the mice from Control and HA groups were infected with *C. rodentium*, and others left uninfected as controls (n = 3–5 mice/group/exp) (**a**). **b**. Hyaluronan-treated mice exhibited less body weight loss than that of *C. rodentium*-infected mice. **c-d**. The fecal bacterial pathogen output and enteropathogen loads in colon in hyaluronan-treated mice were lower than those in *C. rodentium*-infected mice (Two-tailed Student’s t-test). **e**. Colonic length were measured. **f-g**. Histopathological score and pathology analysis of colon showed attenuated histologic damages in hyaluronan-treated mice. **h-i**. Colonic IL-1β and TNF-α expression were determined by qRT-PCR. **p < .01, *p < .05 represent the differences between *Citrobacter*-infected (CB) versus the Control group; ^##^p < .01, ^#^p < .05 show the differences between HA-CB and the CB group. Significance was determined by one-way ANOVA test (Tukey’s multiple comparison test). The data shown are the mean ± the SEM and combined from three independent experiments. **j-n**. In long term experiments, the treatment of hyaluronan in *Citrobacter*-infected mice was prolonged for additional 14 days. Hyaluronan-treated mice gained significantly more body weight (j), reduced enteropathogen loads in colonic tissue (k), thinner and longer colons (l), and faster recovery of colon injury and inflammation (m and n) than those with *C. rodentium* infection alone. The data shown are the mean ± the SEM (n = 3–5 mice/group). ^#^p < .05 versus the PBS+CB group. Significance was determined by a Two-tailed Student’s t-test

To determine the functional significance of the hyaluronan-induced microbiota alterations, the mouse model of *C. rodentium*-associated colitis was used (Figure 2a). BALB/c mice were pre-treated with hyaluronan or PBS daily for 2 weeks and inoculated or not with *C. rodentium*. Mice in hyaluronan group were given hyaluronan once a day during the experimental period. As expected, mice infected with *C. rodentium* showed significant weight loss compared to non-infected mice. In comparison, less body weight loss was observed in hyaluronan-treated and *Citrobacter*-infected mice (Figure 2b), indicating a protective effect of hyaluronan. We next examined whether hyaluronan affected the dynamics of the bacterial infection. Determination of the bacterial pathogen burden in the feces and colonic pathogen loads showed that hyaluronan-treatment resulted in reduced *C. rodentium* colonization during infection and significantly reduced the enteropathogen output in the feces at later stages of infection (Figure 2c). Furthermore, hyaluronan treatment resulted in a significant reduction of *C. rodentium* loads in colonic tissue at 14 days after infection (Figure 2d), whereas the mice with *C. rodentium* infection alone remained heavily infected. Together, these findings demonstrate that pre-treatment with hyaluronan enhanced host control and eradication of *C. rodentium* in the intesitne.

Macroscopic examination of the intestine from *C. rodentium* infected mice showed thicker and shorter colons, however, hyaluronan treatment attenuated these changes (Figure 2e). Our microscopic analysis of colonic tissues showed the development of colonic inflammation in *C. rodentium*-infected mice, as indicated by colonic crypt elongation and hyperplasia, massive cellular infiltration, and mucosal erosion; these histologic damages were found to be significantly attenuated in hyaluronan-treated mice (Figure 2f, g). Correspondingly, significantly lower levels of IL-1β and TNF-α transcripts were detected in hyaluronan-treated mice than those detected in mice with *C. rodentium* infection alone (Figure 2h, i). Taken together, these results demonstrate that pre-treatment with hyaluronan protects against *C. rodentium*-induced colon injury and intestinal inflammation.

To further determine the effects of hyaluronan treatment on host protection against *C. rodentium*-induced colitis in mice, we prolonged the treatment of hyaluronan in *Citrobacter*-infected mice for additional 2 weeks (total 4 weeks post infection). Our results showed that hyaluronan-treated mice gained significantly more body weight than PBS-fed control (Figure 2j) and had reduced enteropathogen loads in colonic tissue at 28 days after infection (Figure 2k). Macroscopic and microscopic analysis showed thinner and longer colons (Figure 2l), and an attenuated colitis in hyaluronan-treated mice (Figure 2m, n), as indicated by relieved colonic crypt elongation and hyperplasia, milder cellular infiltration, relatively intact intestinal mucosa, and decreased inflammatory score compared with *C. rodentium* infected mice alone, suggesting the hyaluronan-treatment promoted the recovery process of bacterial colitis. Our collective data suggest that hyaluronan not only attenuates *C. rodentium*-induced colitis in mice but also promotes the recovery process.

### Hyaluronan treatment increases resistance of mice to DSS treatment and results in attenuated intestinal inflammation

**Figure 3.**
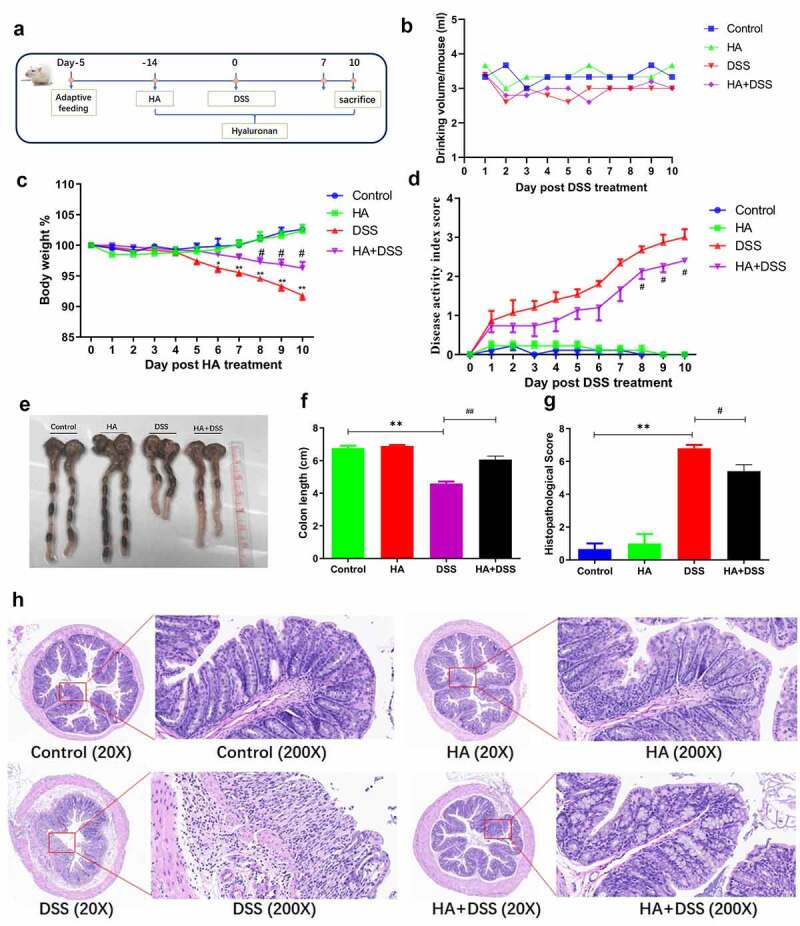
**Hyaluronan treatment increases resistance of mice to DSS treatment and results in attenuated intestinal inflammation**. Balb/c mice were orally gavaged with hyaluronan or PBS once a day for two weeks, some of the mice were given drinking water containing 2.5% (w/v) DSS ad libitum for 7 days, followed by distilled water for 3 days, and others left untreated as controls (**a**). **b**: Dynamic changes of the daily water intake of each mice was recorded and show no significant difference among groups. **c-e**. DSS treatment resulted in a significant increase in colonic inflammation, characterized by developed body weight loss (c), and a significantly increased level of disease activity index (DAI, rectal bleeding and diarrhea) (d), and hyaluronan administration alleviated the observed changes. **e, f**: Macroscopic examination of the intestine from HA-treated colitis mice showed attenuated shortening and thickening of colons. **g, h**: Microscopic examination revealed an attenuated mucosal damages and inflammatory cell infiltration in hyaluronan-treated mice. N = 3–5 mice/group. **p < .01, *p < .05 versus the Control group; ^##^p < .01, ^#^p < .05 versus the DSS group. Significance was determined by one-way ANOVA test (Tukey’s multiple comparison test)

To further evaluate the effects of hyaluronan-treatment on intestinal homeostasis in BALB/c mice, hyaluronan or PBS treated mice were given drinking water containing 2.5% (w/v) dextran sodium sulfate (DSS) ad libitum for 7 days, and the growth performance and the induction and progression of colitis were evaluated (Figure 3a). We recorded the daily water intake of each mouse and did not find any differences among them, especially between the DSS and HA+DSS groups, suggesting similar exposure to DSS in the intestinal mucosa in both groups of mice (Figure 3b). We then evaluated the clinical phenotypes of control and DSS-treated mice. As anticipated, we found that DSS treatment resulted in a significant increase in colonic inflammation, characterized by body weight loss, rectal bleeding, and diarrhea, leading to a significantly increased level of disease activity index (DAI) (Figure 3c, d). In contrast, hyaluronan administration alleviated the changes caused by DSS treatment (Figure 3c, d). Macroscopic examination of the intestine from DSS-treated mice showed thicker and shorter colons compared to the control mice. However, supplementation of hyaluronan attenuated shortening and thickening of colon in DSS-induced mice (Figure 3e, f). Microscopic examination revealed that mice with DSS-induced colitis showed typical pathological changes, including damaged crypts and epithelial integrity, remarkable inflammatory cell infiltration in the mucosa, and increased histopathological score, but hyaluronan-administration attenuated those effects in DSS-treated mice (Figure 3h). These results support the protective role of hyaluronan in the murine DSS-colitis model.

Taken together, our results from both bacterial- and DSS-induced colitis models demonstrate that hyaluronan supplementation attenuates intestinal inflammation in both cases of exogenous bacterial infection and mucosal chemical damage. These results suggest a functional role of hyaluronan in maintaining intestinal homeostasis.

### *Hyaluronan modulates immune responses and epithelial barrier properties during* C. rodentium *infection*

**Figure 4.**
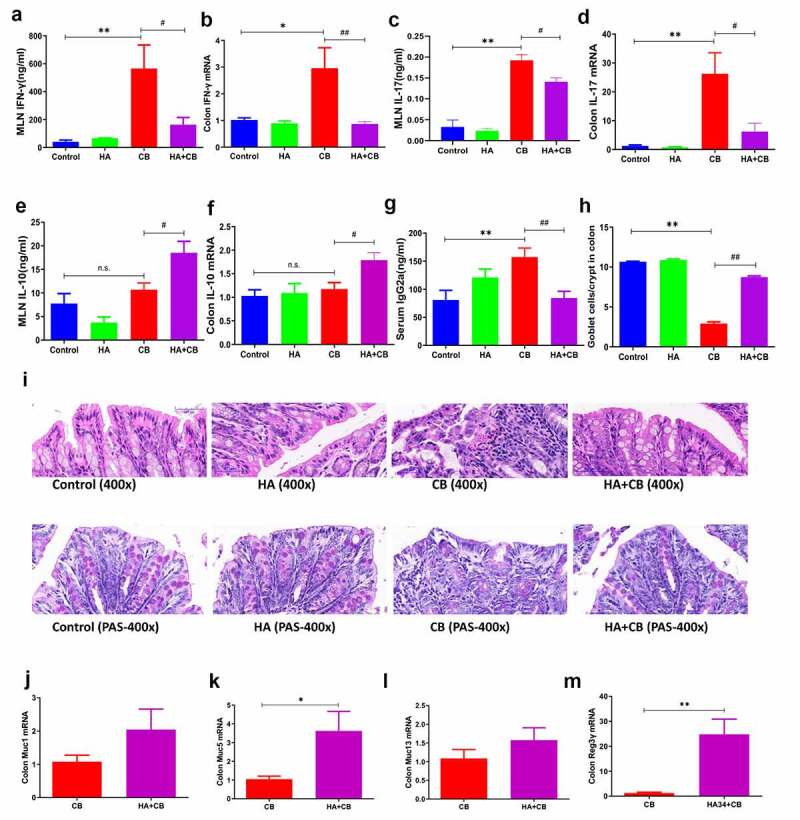
**Hyaluronan modulates immune responses and epithelial barrier properties during *C. rodentium* infection. a-c**. Mesenteric lymph nodes (MLNs) cells were collected from the mice, stimulated in vitro with anti-CD3 mAb, and culture supernatants were collected 72 h later. Cytokines (IFN-γ, IL-17 and IL-10) secretion into the culture supernatants were determined. **d-f**. Colon tissues were collected and IFN-γ, IL-17 and IL-10 mRNA expression was determined by qRT-PCR. Data show the fold changes of mRNA expression level of experimental groups compared to baseline obtained from control mice **g**. Serum IgG2 levels were measured by Elisa. **h-i**. Goblet cells were stained with periodic acid-schiff (PAS), and the number of goblet cells per crypt in the colon were quantified. **j-k**. qRT-PCR analysis of colon showed that hyaluronan treatment resulted in a significant increase of mucus, such as muc1, muc5 and muc13 during *C. rodentium* infection. **m**. mRNA expression of Regenerating Islet-Derived 3 (Reg3)γ. N = 3–5 mice/group/exp, **p < .01, *p < .05 versus the Control group; ^##^p < .01, ^#^p < .05 versus the CB group, n.s. = no significant. Significance was determined by one-way ANOVA test (Tukey’s multiple comparison test). The data shown are the mean ± the SEM and combined from two independent experiments

Emerging evidence has shown a strong link between *C. rodentium* infection and induction of Th1 adaptive immune response.^18^ To examine the influence of hyaluronan treatment on an ongoing Th1 immune response, mesenteric lymph node (MLN) cells were collected and restimulated *ex vivo* by anti-CD3 monoclonal antibody. Cytokine production was analyzed using ELISA. As expected, a highly Th1 polarized response was detected in the mucosal immune compartments in mice with *C. rodentium* infection, evidenced by the detection of increased production of IFN-γ. In contrast, the level of IFN-γ was significantly lower in hyaluronan-treated mice with *C. rodentium* infection (Figure 4a). Our qRT-PCR analysis of the colon tissues further confirmed that hyaluronan had an obvious inhibitory effect on IFN-γ production in *C. rodentium*-infected mice (Figure 4b), suggesting that hyaluronan can significantly inhibit the bacterium-induced Th1 response. This conclusion was further confirmed by analysis of the serum antigen-specific IgG2a, an important antibody indicator of Th1 immune response, which was enhanced by *C. rodentium* infection and reduced by hyaluronan treatment (Figure 4g). Moreover, *C. rodentium* infection also causes an upregulation of the Th17 response.^19^ ELISA results from MLN cells and qRT-PCR analysis of the colon both revealed elevated levels of IL-17 in mice infected with *C. rodentium* (Figure 4c, d). Interestingly, a significant reduction of IL-17 production was detected with hyaluronan treatment and *C. rodentium* infection. Moreover, our results showed that the hyaluronan-associated suppression of Th1/Th17 response in *C. rodentium*-infected mice correlated with an elevated response of IL-10, an immune suppressive cytokine associated with T regulatory cells, in the MLN cells and colons from *C. rodentium*-infected BALB/c mice treated with hyaluronan (Figure 4e, f).

The intestinal mucus layer, an important element of epithelial protection and suppression of inflammatory activity, is produced by goblet cells and plays an important role in protection against different type of injuries.^20^ Therefore, goblet cell dysfunction results in reduced secretion of mucus and decreases host’s ability to defend against pathogens. To determine whether hyaluronan could affect colonic goblet cell response, contributing to the observed protection, Periodic Acid Schiff (PAS) staining on the colonic sections from each mice was performed. Our results showed a significant decrease in the number of goblet cells (PAS+ cells) in the colons of *C. rodentium*-infected mice compared to the control mice, and hyaluronan-treatment resulted in significant increase in goblet cell numbers per crypt in *C. rodentium*-infected mice (Figure 4h and i). Consistently, our qRT-PCR analysis further showed that hyaluronan treatment resulted in an increased mRNA expression of muc1, muc5, and muc13 during *C. rodentium* infection (Figure 4j-l). These observations suggest that one of the potential mechanisms by which hyaluronan protects mice against *C. rodentium* infection may involve enhanced mucus barrier function.

To elucidate further the mechanism by which hyaluronan exerts protective effect on intestinal mucosa, we determined if hyaluronan treatment influences the innate mucosal antimicrobial peptide (AMP) responses by examining mRNA expression of AMPs. Our results from qRT-PCR analysis revealed a remarkable increase of colonic expression of Regenerating Islet-Derived 3 (Reg3)γ, which has been shown to play protective roles in *C. rodentium* infection,^21^ in hyaluronan-treated and *C. rodentium*-infected mice (Figure 4m). Taken together, these results show that HA treatment reduces intestinal inflammation as a result of enhanced eradication of *Citrobacter*, and that the attenuated colitis is reflected by the preservation of goblet cells and innate host defense. These results provide evidence to demonstrate a role for hyaluronan in the regulation of innate host defense as well as immune regulatory response and the modulation of intestinal barrier function, contributing to the inhibition of *C. rodentium* colonization/infection and the attenuation of bacteria-induced intestinal inflammation.

### *Hyaluronan supplementation partially attenuates* C. rodentium*-induced gut dysbiosis*

**Figure 5.**
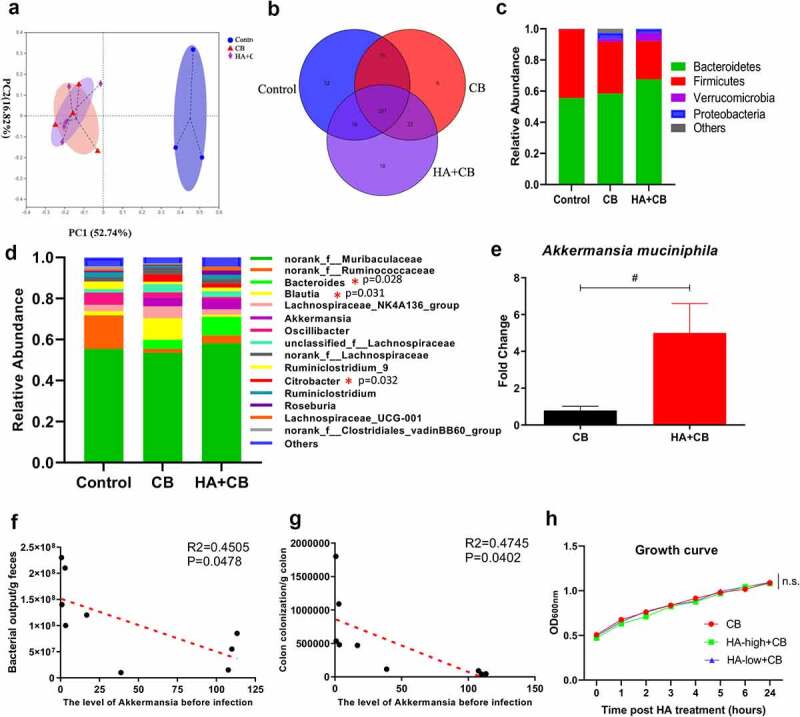
**Hyaluronan supplementation attenuated *C. rodentium*-induced gut dysbiosis**. Fecal samples (n = 3–5 mice/group) were collected for microbiome profile analysis by using bacterial 16S rRNA gene sequencing. **a**. PCoA analysis showed a significant separation between the Control and *C. rodentium*-infected groups; **b**. Venn diagram. Relative abundances of bacterial groups at the phylum (**c**) and genus (**d**) levels, and statistical significance among groups at the genus levels, tested by means of Kruskal–Wallis H test. **e**. Comparison of relative abundance genus *Akkermansia* between PBS- and hyaluronan-treated mice with *C. rodentium* infection (^#^p < .05 versus the CB group, Two-tailed Student’s t-test). Pearson correlation analyses between the level of *A. muciniphila* before infection and pathogen bacterial output (**f**), and enteropathogen tissues loads (**g**) of corresponding mice with *C. rodentium* infection. **h**. The growth curve of *C. rodentium* with or without hyaluronan treatment *in vitro* bacterial culture and showed that the growth of *C. rodentium* was not affected by hyaluronan treatment. (Data are shown as mean ± SEM, n = 10/group for each time point, n.s. = not significant, one-way ANOVA test)

To better understand whether and how hyaluronan modulates the complex interface between microbiota and mucosal immunity in the context of enteropathogen infection, we determined fecal microbiota structures and compositions in mice. In a separate sequencing analysis, a total of 607,099 raw reads and an average of (50,591.6 ± 3,284) reads per sample were obtained. PCoA analysis showed a significant separation between the control and *C. rodentium*-infected groups. However, no significant difference was detected among the mice infected with *C. rodentium* (Figure 5a). Venn diagram revealed that 201 OTUs overlapped among the three groups, and 223 OTUs in the *C. rodentium*-infected mice with PBS and hyaluronan (Figure 5b). The results from taxonomic composition analysis showed a clear difference in microbiota composition among the three groups at the phylum level, which is evidenced by the results showing a decreased abundance in Firmicutes, and an increase in Bacteroides, Verrucomicrobia, and Proteobacteria in *C. rodentium* infected mice with the changes being more pronounced in *Citrobacter*-infected mice that received hyaluronan treatment (Figure 5c). Changes in the main microbiota at the genus level are shown in Figure 5d. The relative abundance of *Bacteroides, Blautia*, and *Lachnospiraceae_NK4A136_group* were increased, and the levels of *Oscillibacter, Ruminiclostridium_9, Ruminiclostridium* and *Roseburia* were decreased due to *C. rodentium* infection (Figure 5d). Interestingly, however, our results showed that *Citrobacter*-induced dysbiosis was partially attenuated in hyaluronan-treated mice. Among the top 15 genus, 11 of them in *Citrobacter*-infected mice were found to be restored or partially restored to the levels similar to that detected in control mice (Figure 5d). Moreover, the relative abundance of *Akkermansia* was higher in hyaluronan-treated and *C. rodentium*-infected mice than that of mice without hyaluronan treatment. Our qPCR analysis further confirmed that hyaluronan treatment led to a preservation of the high level of *Akkermansia* in the hyaluronan-treated mice prior to *Citrobacter* infection (Figure 5e). Pearson correlation analyses (figure 5f, g) showed that the levels of *A. muciniphila* before *C. rodentium* infection were negatively related to the enteropathogen bacterial output and enteropathogen tissue loads of corresponding mice with *C. rodentium* infection. These observations further demonstrated the role of hyaluronan in the modulation of gut microbiome composition, preferentially in promoting *A. muciniphila* bacteria. Moreover, our microbiota analysis also revealed that the relative abundance of *Citrobacter* in the hyaluronan-treated mice was significantly lower than that in mice infected by *C. rodentium* alone (Figure 5d), which was consistent with our results of described above (Figure 2c, d) showing a reduced fecal bacterial pathogen output and enteropathogen loads in colonic tissue. To determine whether hyaluronan has a direct and significant inhibitory effect on the growth of *C. rodentium*, our next set of *in vitro* experiments generated a growth curve for liquid culture of *C. rodentium* in the presence and absence of hyaluronan. As shown in Figure 5h, the growth of *C. rodentium* was not affected by hyaluronan treatment, suggesting strongly that the mechanism by which hyaluronan reversed *C. rodentium*-induced bacterial colitis was possibly via its effects on the host gut microbiota composition and functionality and/or host immune system rather than via direct killing of *C. rodentium*.

### The protective effects of hyaluronan are transferable by fecal transplantation

**Figure 6.**
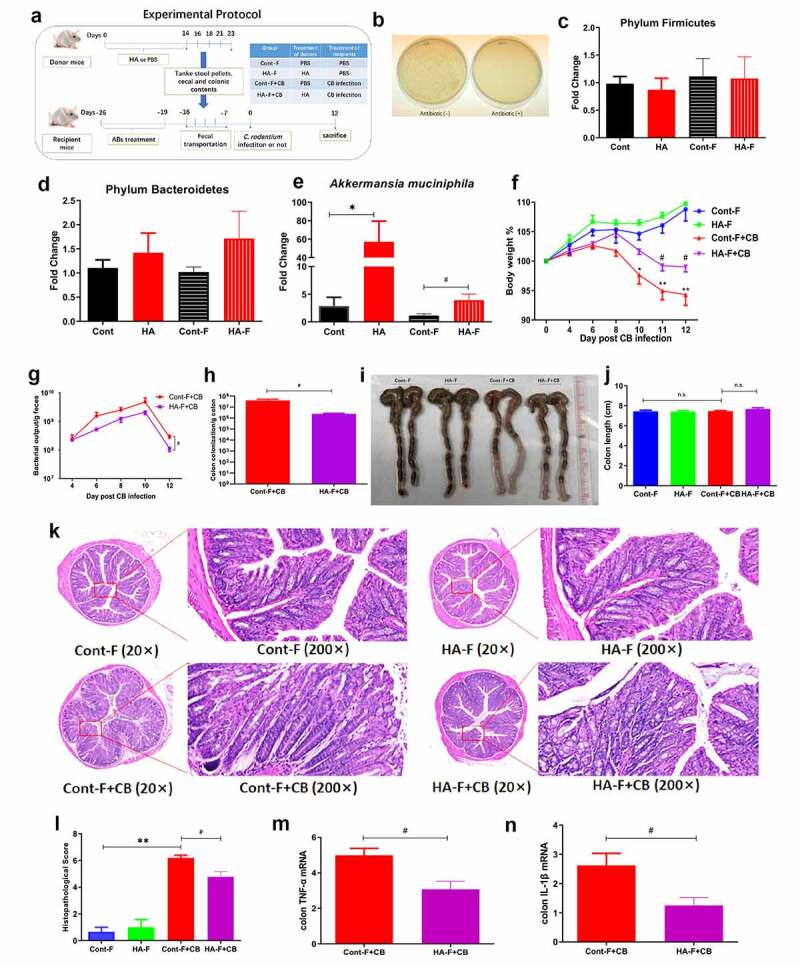
**Microbiota transplantation from hyaluronan-treated mice recapitulated the effects of hyaluronan on *C. rodentium*–induced colitis mice. a**. Microbiota transplantation protocol. The gut microbiota (mixture of stool pellets, cecal and colonic contents) were collected from donors and orally gavaged to the corresponding recipients pretreated with a 7-day antibiotic cocktail. Some of the recipients were infected with *C. rodentium*, and others left uninfected as controls. **b**. Effects of antibiotic on gut bacterial growth in mice. **c-e**. qPCR results showed a similar pattern of the representative of gut microbial distribution between the donors and the recipients. Data show the fold changes of mRNA expression level of experimental groups compared to baseline obtained from control mice. **f**. Recipient mice that were colonized with microbiota from HA-treated donors and infected with *C. rodentium* (HA-F+ CB) exhibited less body weight loss than that of recipient mice that are colonized with microbiota from control donor and infected with *Citrobacter* (Cont-F+ CB). **g-h**. The fecal bacterial pathogen output and colonic enteropathogen loads in HA-F+ CB group were lower than those in Cont-F+ CB group (*p < .05, Two-tailed Student’s t-test). **i, j**. Colon length measured after sacrifice. **k-l**. Pathology analysis and histopathological score of colon showed less colonic epithelial damage and decreased cellular infiltration of the lamina propria in mice colonized with hyaluronan-induced microbiota and followed with *C. rodentium* infection (HA-F+ CB) compared to Cont-F+ CB group. **m-n**. qRT-PCR analysis of colonic tissues showed a significant decrease of TNF-α and IL-1β levels in *C. rodentium*-infected recipients of microbiota from hyaluronan-treated donors (HA-F+ CB). Data was shown as mean ± SEM (n = 3–5 mice/group/exp). **p < .01, *p < .05 versus the Cont-F group; ^##^p < .01, ^#^p < .05 versus the Cont-F+ CB group; n.s. = not significant. Significance was determined by one-way ANOVA test (Tukey’s multiple comparison test). Data were combined from two independent experiments

To investigate whether the hyaluronan-induced gut microbiome was necessary and sufficient to protect mice against *C. rodentium* infection, we utilized the fecal microbiota transplantation approach (Figure 6a). We first confirmed the effective depletion of the gut microbiota by a week-long antibiotic treatment, which was supported by the results showing no culturable bacterial growth in overnight fecal culture of antibiotic-treated recipient mice (Figure 6b). The recipients were then gavaged with the gut microbiota (mixture of stool pellets, cecal, and colonic contents) 5 times over the course of two weeks from control or hyaluronan-treated donors. Our results revealed the high similarity in the pattern of the microbiota community between the control or hyaluronan-treated donors and the corresponding recipients, as evidenced by the similar levels of phylum Firmicutes and Bacteroides, and increased *A. muciniphila* in hyaluronan-treated donor mice and mice that were colonized with hyaluronan-altered microbiota compared to the corresponding control mice (Figure 6c-e).

We next investigated the functional significance of hyaluronan-induced gut microbiome in the modulation of host response to *C. rodentium* infection. We found that the mice that received microbiota from hyaluronan-treated donors showed resistance to *C. rodentium* infection. This is evidenced by attenuated weight loss (Figure 6f), reduced fecal bacterial pathogen output (Figure 6g) and enteropathogen loads in colon (Figure 6h) compared to *Citrobacter*-infected recipients of control microbiota. Moreover, macroscopic examination of the colon showed that colonic thickening was less pronounced in the recipients of microbiota from hyaluronan-treated donors, although their length didn’t have any obvious difference (Figure 6i, j). H-E staining of colonic tissues further revealed that mice colonized with hyaluronan-induced microbiota and followed with *C. rodentium* infection exhibited less colonic epithelial cell hyperplasia and crypt elongation, decreased cellular infiltration of the lamina propria and epithelial erosions (Figure 6k). The severity of *C. rodentium*-induced tissue damage was significantly attenuated in *C. rodentium*-infected recipient mice that were colonized with hyaluronan-induced microbiota, as evidenced by a lower inflammatory score (Figure 6l). Correspondingly, our qRT-PCR analysis of colonic tissues showed a significant decrease of IL-1β and TNF-α levels in *C. rodentium*-infected recipients of microbiota from hyaluronan-treated donors relative to *C. rodentium*-infected recipients of control microbiota (Figure 6m, n). Collectively, these results demonstrate that microbiota transplantation from hyaluronan-treated mice faithfully recapitulated the protective effects of hyaluronan treatment on *C. rodentium*–induced colitis.

### *Hyaluronan-induced enrichment of* A. muciniphila *alleviates bacterial colitis in mice*

**Figure 7.**
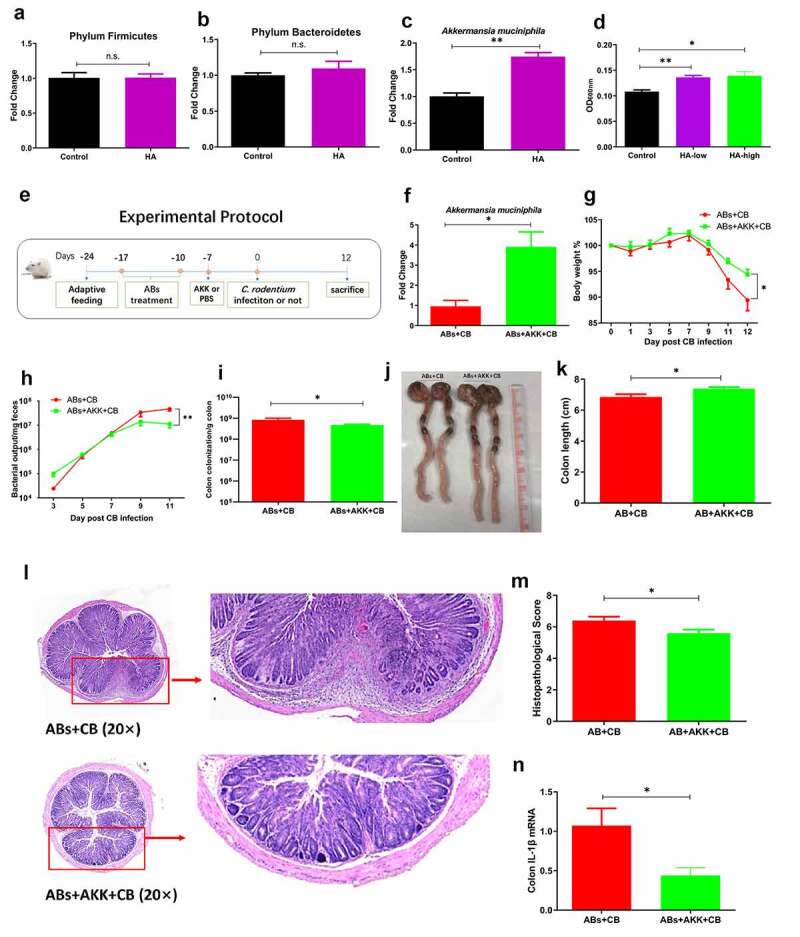
**Hyaluronan-mediated enrichment of *A. muciniphila* alleviated *C. rodentium*-induced bacterial colitis in mice**. Gut microbiota-depleted mice were gavaged daily with *A. muciniphila*, infected with *C. rodentium* 7 days after *A. muciniphila* inoculation, and followed by examination of bacterial colitis-related traits. **a-c**. Fresh fecal samples from control mice were collected to prepare 20% (w/v) fecal slurry. The relative abundance of Firmicutes (a), Bacteroidetes (b), and *A. muciniphila* (c) were measured by qPCR (data show the fold changes of experimental groups compared to baseline obtained from control mice). The level of *A. muciniphila* was determined by measuring bacterial density at 600 nm after 72 hours incubation under anaerobic conditions by fecal batch culture fermentation *in vitro* (**d**). In *vitro* bacterial cultures of *A. muciniphila* with hyaluronan treatment showed that hyaluronan has a direct and significant growth promoting effect on the growth of *A. muciniphila*. **e**. Experimental protocol. **f**. Detection of *A. muciniphila* colonization. **g**. *A. muciniphila* treated-mice exhibited less body weight loss than that of PBS-treated mice with *C. rodentium* infection. **h, i**. Colonization of mice with *A. muciniphila* resulted in decreased fecal *C. rodentium* output (h) and enteropathogen loads in colon (i). **j, k**. Colonic tissues and tissue length. **l, m**. Microscopic examination revealed that mice colonized with *A. muciniphila* and infected with *C. rodentium* showed attenuated *C. rodentium-*induced pathological changes, and lower pathology scores (m). **N**. qRT-PCR analysis of colonic tissues showed a significant lower level of IL-1β response in *C. rodentium*-infected mice with *A. muciniphila* colonization. Data was shown as mean ± SEM (n = 3–5 mice/group/exp). **p < .01, *p < .05 versus the ABs+CB group. Significance was determined by a Two-tailed Student’s t-test. Data were combined from two independent experiments

The microbiota transfer experiments demonstrated the functional significance of hyaluronan-induced microbiome in host protection against *C. rodentium* infection. Microbiota analysis described above identified *A. muciniphila* to be the key species responding to hyaluronan treatment, and the level of *A. muciniphila* was negatively related to the bacterial output and enteropathogen tissues loads of corresponding mice with *C. rodentium* infection. We then hypothesized that hyaluronan may exert the beneficial effects by acting as a prebiotic to enrich the commensal bacterium *A. muciniphila*. To test this hypothesis, we first assessed the direct effects of hyaluronan treatment on *A. muciniphila*. We collected and cultured feces from the control mice. The relative abundance of *A. muciniphila* was measured after 72 hours incubation with or without hyaluronan under anaerobic conditions. Our results from qPCR analysis revealed hyaluronan enriched has no appreciable effects on the levels of Bacteroidetes and Firmicutes (Figure 7a, b, but significantly *A. muciniphila* (Figure 7c), which was consistent with our results of *in vivo* studies described above (Figure 1h). This observation was confirmed by our results of *in vitro A. muciniphila* culture experiment, in which *A. muciniphila* were cultured with or without hyaluronan, showing that hyaluronan has a direct and significant growth promoting effect on the growth of *A. muciniphila* (Figure 7d).

To formally demonstrate the functional significance of hyaluronan-enriched *A. muciniphila* in the modulation of host response to bacterial enteropathogen infection and intestinal inflammation, gut microbiota-depleted mice were gavaged daily with *A. muciniphila* for 7 days and infected with *C. rodentium*. Bacterial colitis-related traits were examined, as illustrated in Figure 7e. Our qPCR analysis of fecal material confirmed that *A. muciniphila* colonization of antibiotic-treated mice resulted in increased detection of *A. muciniphila* (Figure 7f). Furthermore, our results showed that colonization of mice with *A. muciniphila* resulted in a significant attenuation of *C. rodentium* infection and the disease it induces in mice. This is evidenced by the observations showing that oral administration of *A. muciniphila* significantly reduced body weight loss in *C. rodentium*-infected mice (Figure 7g), which correlated with reduced fecal bacterial pathogen output and enteropathogen tissue loads, compared to *C. rodentium*-infected mice treated with PBS (Figure 7h, i). Macroscopic analysis of colonic tissues of *A. muciniphila*-colonized mice following *C. rodentium* infection revealed a less pronounced colonic thickening and shorter length of colon (Figure 7j, k). Microscopic examination revealed that mice colonized with *A. muciniphila* and infected with *C. rodentium* showed attenuated *C. rodentium-*induced pathological changes, including mild to moderate cellular infiltration in the lamina propria and epithelial erosions, and lower pathology scores relative to the mice infected only with *C. rodentium* (Figure 7l, m). Furthermore, our qRT-PCR analysis of colonic tissues showed a significant lower level of proinflammatory cytokine (IL-1β) response in *C. rodentium*-infected mice with *A. muciniphila* colonization than that detected in mice with *C. rodentium* infection alone (Figure 7n). These *A. muciniphila-*induced protective effects in mice infected with the enteropathogen are consistent with our results described above showing the protection in *C. rodentium-*infected hosts that were treated with hyaluronan or transferred with hyaluronan-induced microbiota (Fig. 2 and 5). Our collective data, therefore, demonstrated that hyaluronan enriched the proliferation of commensal bacterium *A. muciniphila*, which plays a crucial and central role in protection against *C. rodentium* infection and intestinal inflammation.

### *The protective effects of* A. muciniphila *on* C. rodentium *induced-colitis are associated with enhanced mucus barrier and anti-microbial responses*

**Figure 8.**
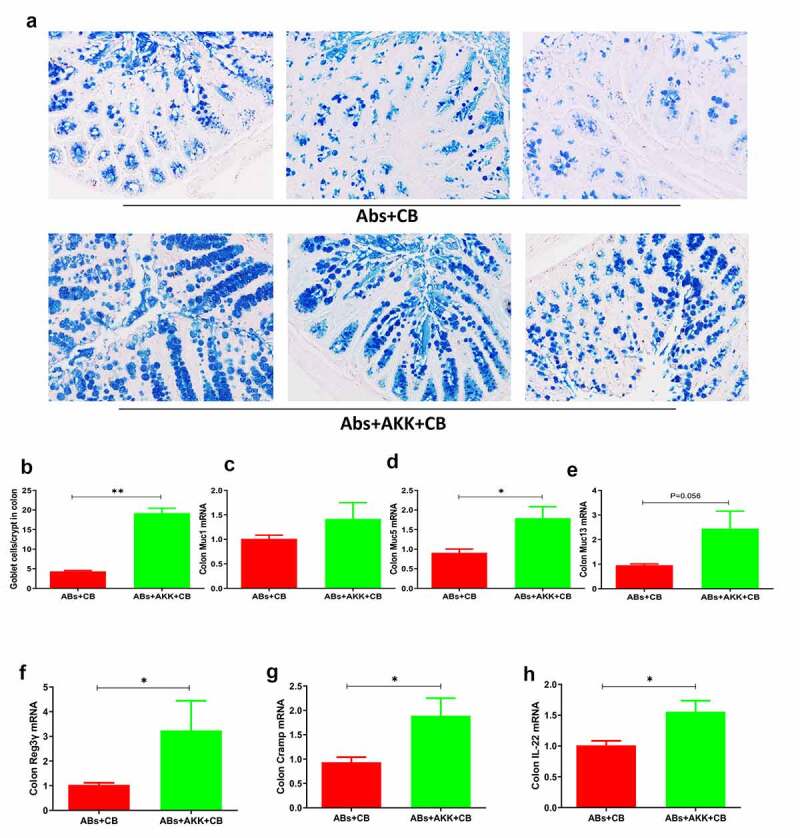
**Mice treated with *A. muciniphila* had enhanced mucus barrier and anti-microbial responses. a, b**. Gut microbiota-depleted mice were gavaged daily with or without *A. muciniphila*, infected with *C. rodentium* 7 days after *A. muciniphila* inoculation. Colonic tissues were collected and sectioned. Goblet cells were stained with AB/PAS and the number of goblet cells per crypt in the colon were quantified. **c-e**. qRT-PCR analysis of colon showed that *A. muciniphila* colonization promotes gene expression of muc1, muc5 and muc13 during *C. rodentium* infection. **f-h**. mRNA expressions of Reg3γ, Cramp, and IL-22 was assayed by qRT-PCR. Data show the fold changes of mRNA levels of antibiotic-pretreated mice that were colonized with *A. muciniphila* and infected with *C. rodentium* (Abs+AKK+CB) compared to baseline obtained from control mice without *A. muciniphila* inoculation (ABs+CB). Data was shown as mean ± SEM (n = 3–5 mice/group/exp). **p < .01, *p < .05. Significance was determined by a Two-tailed Student’s t-test. Data were combined from two independent experiments

Our previous results have shown that hyaluronan-induced alterations of the gut microbiome, especially enrichment of *A. muciniphila*, protects mice against *C. rodentium* infection and intestinal inflammation, through a mechanism that involves the preservation of goblet cells, which produce mucins, and induction of AMPs. To determine if this mechanism holds true in mice colonized with *A. muciniphila*, we next determined whether colonization of mice with *A. muciniphila* promotes goblet cell responses to *C. rodentium*. The data from AB/PAS staining indicated that the *A. muciniphila*-colonized, *Citrobacter*-infected mice displayed markedly increased numbers of goblet cells in colons compared to the mice infected with *C. rodentium* alone (Figure 8a). In line with this observation, the results from qRT-PCR analysis showed that colonization of mice with *A. muciniphila* resulted in upregulated expressions of gene encoding mucin, including muc1, muc5, and muc13 (Figure 8b-e).

To further clarify how *A. muciniphila* protects against *C. rodentium* infection, gene expressions of AMPs in the colon were examined and the results revealed that mice colonized with *A. muciniphila* and infected with *C. rodentium* displayed significant upregulation of Reg3γ and CRAMP, both of which have been shown to protect against *C. rodentium* infection,^22^ relative to the mice infected only with *C. rodentium* (Figure 8f, g). IL-22 has been suggested to increase the expressions of mucus and antimicrobial proteins and help prevent dissemination of enteric pathogenic bacteria.^23^ In this study, qRT-PCR analysis of colonic tissue revealed that colonization of mice with *A. muciniphila* resulted in up-regulated IL-22 expression during *Citrobacter* infection (Figure 8h). These results suggest that one of the mechanisms underlying the protective effects of *A. muciniphila* on *C. rodentium* induced colitis involved regulation of the mucus barrier and AMPs in the gut. These *A. muciniphila-*induced protective mechanisms on *C. rodentium-*infected mice are consistent with our results described above showing hyaluronan-enhanced mucus barrier and anti-microbial responses (Figure 3), leading to the enhanced eradication of *Citrobacter* and attenuation of intestinal inflammation.

## DISCUSSION

Recent evidence has demonstrated the beneficial effects of hyaluronan in several medical conditions, including intestinal inflammatory disorders.^11,12^ However, the exact mechanisms by which hyaluronan exerts the protective effects on intestinal epithelium are not fully understood. One important and unanswered question is whether orally delivered hyaluronan affects gut microbiota, contributing to enhanced intestinal epithelial defense and improved intestinal health. Our current study showed that hyaluronan treatment not only induces alterations in the gut microbiota composition in mice, but also protects mice against both pathogen- and chemically induced intestinal damage. We showed that microbiota transplantation from hyaluronan-treated mice recapitulates the effect of hyaluronan on *Citrobacter* infection and bacterial colitis. More significantly, our results for the first time, demonstrate that colonization of mice with *A. muciniphila*, a commensal bacteria that is found to be selectively enriched by hyaluronan treatment in our current study, is sufficient to recapitulate the protective effects of hyaluronan and hyaluronan-altered microbiota on bacterial infection and intestinal inflammation. These results, therefore, suggest a functional role for the hyaluronan-induced microbiota, particularly hyaluronan-enriched commensal bacterium *A. muciniphila*, in the modulation of host resistance to bacterial enteropathogen infection and intestinal inflammation. The results from our current investigation suggest a novel mechanism for hyaluronan-induced protection in intestinal mucosa.

The gut mucosal barrier, constituted by the epithelium, the microbiota, and the immune cells that are resident in or recruited to the sub-epithelial mucosa, is critical in effectively controlling enteropathogens and maintaining intestinal epithelial homeostasis. Although evidence suggests that hyaluronan plays roles in normal intestinal tissue homeostasis and innate host defense mechanisms,^24^ the interactive link between hyaluronan and the microbiota, one of the major mucosal barrier components, is not fully understood, especially in the context of enteric bacterial infection. Work from our laboratory and others has shown that the modulation of the intestinal microbiome by diet, nutritional supplementation, drugs or intestinal helminth infection could alter host resistance to enteric *C. rodentium* and the severity of *C. rodentium*-induced intestinal inflammation in mice.^25,26^ These observations suggest that maintenance of the homeostasis of intestinal microbiome, the largest symbiotic ecosystem of the host, is likely to play an irreplaceable role in host resistance and immune function against pathogen infection.

The current mechanistic investigation determined whether hyaluronan treatment may alter intestinal microbiome composition and functionality, contributing to enhanced host resistance against intestinal bacterial pathogens. By utilizing bacterial 16S rRNA gene sequencing and *Citrobacter*-infection approaches, we formally determined the contribution of the hyaluronan treatment to host fecal microbial communities and host defense against enteropathogen infection. Our results revealed that hyaluronan treatment of BALB/c mice induced an increase of intestinal microbiota diversity and a significant alteration in the gut microbiome, especially enrichment of the commensal bacterium *A. muciniphila*. However, a previously published human study, which focused on the evaluation of the safety of hyaluronan in human subjects, indicated that human subjects who received oral hyaluronan (140 mg in water once daily for 7 days) showed no difference in the most abundant microbiome taxa at phylum and family levels.^27^ This discrepancy with our findings may be related to the differences in the host (human vs mice), the experimental approaches (course and dosage of hyaluronan treatment and data analysis approaches) and the potential differences in the gut microbiota sensitivity to hyaluronan between human and mice. To directly determine the functional consequences of hyaluronan-induced microbiota in regulating host protection against bacterial enteropathogens, microbiota transfer experiments were performed in the current investigation. Our results demonstrated that transfer of hyaluronan-induced gut microbiome is sufficient to recapitulate the protective effect of hyaluronan treatment on host resistance to *C. rodentium* infection, demonstrating the protective and functional role for hyaluronan-induced microbiota in *C. rodentium*-infection and bacterial colitis. The results from this current study demonstrate that hyaluronan protects against *C. rodentium* infection and intestinal inflammation in mice by modulating host gut microbiota composition and functionality.

Our subsequent experiments examined how exactly hyaluronan-altered gut microbiome exerts the protective effects. In our study, *A. muciniphila* has been identified to be the key bacterial species responding to hyaluronan treatment, which is manifested in the synchronous increase in hyaluronan-treated mice with and without *C. rodentium* infection, and that the abundance of *A. muciniphila* is negatively correlated with *Citrobacter* infection and colitis in mice. We speculated that the protective effect of hyaluronan treatment is highly likely due to enrichment of the commensal bacterium *A. muciniphila*. This idea is strongly supported by our results of *A. muciniphila* intervention experiments, showing an enhanced resistance to *Citrobacter* infection and intestinal inflammation in mice inoculated with *A. muciniphila. A. muciniphila*, a gram-negative and strictly anaerobic bacterium, is the only cultivated intestinal representative of the phylum Verrucomicrobia^28^ and has been implicated in gut barrier function, immune response, and host metabolism^29,30.^ Notably, it has been suggested that reduced levels of *A. muciniphila* have been observed in patients with obesity,^31^ diabetes,^32^ metabolic disorders,^33^ and inflammatory bowel diseases (mainly ulcerative colitis),^34^ which suggests it may have potential role to play in regulating host functions, anti-inflammatory properties and protection against enteropathogenic bacteria infection. Despite these effects, it is unclear whether and how *A. muciniphila* affects host immune and inflammatory responses against bacterial enteropathogens. By colonization of mice with *A. muciniphila*, we show the protective role of this commensal in *C. rodentium* infection. However, *A. muciniphila*, a mucin degrader, has also been linked to impaired protection from *C. rodentium* when mice are on a fiber free diet.^35^ In that study it was shown that in the absence of dietary fiber, the mucin-degrading bacteria and the thin mucus layer promoted *Citrobacter*-infection. The intestinal mucus layer is produced by goblet cells and plays an important role in the protection of intestine against different type of injuries. Mice genetically deficient in mucin production (Muc2KO) are highly susceptible to *C. rodentium* infection.^36^ Our experimental results show that colonization of *A. muciniphila* in mice (on normal diet) results in increased goblet cells and mucin production in *Citrobacter*-infected mice and these mice are protected from disease, suggesting that one of the mechanisms underlying the protective effects of *A. muciniphila* on *C. rodentium* infection and colitis involved regulation of the mucus barrier in the gut. In addition, our results provide evidence to indicate *A. muciniphila* colonization results in significant upregulation of Reg3γ and CRAMP, both of which have been shown to protect against *C. rodentium* infection.^22^ Together, these results suggest that hyaluronan treatment enhanced intestinal mucus barrier function and anti-microbial responses during bacterial enteropathogen infection, leading to the suppression of *C. rodentium* colonization and intestinal inflammation. The results from the current study provide strong evidence to suggest that hyaluronan treatment specifically promotes the growth of *A. muciniphila* and that hyaluronan may be used as a specific prebiotic of *A. muciniphila* to promote mucosal defense and intestinal health. Therefore, target-specific microbial species may have unique therapeutic promise for various diseases. Taken together, our results provided novel insights into the consequences of hyaluronan supplementation by identifying and functionally characterizing the hyaluronan-induced gut microbial communities, particularly, hyaluronan-enriched commensal bacterium *A. muciniphila* that influence host mucosal inflammatory and immune responses to enteropathogen bacterial infection. These results will aid in developing novel microbiota-based strategies, i.e., ones based on altering microbiota composition with pre-, pro-, and synbiotics, for the prevention and treatment of various disease conditions, including patients who may have intestinal microbiota dysbiosis and who are at increased risk for enteric bacterial infections.

Our results also provide strong evidence to suggest that one of the potential mechanisms by which hyaluronan ameliorates *C. rodentium*-induced colitis may be through the reprogramming of gut microbiota in mice. Previous study found that *C. rodentium* infection led to its gut overgrowth at levels that took up 1–3% of the total intestinal microbiota with 10^9^ cfu per g in the colon.^37^ Our results showed that hyaluronan treatment can partially alleviate *C. rodentium*-induced dysbiosis of gut microbiome, accompanied by a significant decrease of the relative abundance of *Citrobacter*. It is especially notable that inclusion of hyaluronan in bacterial culture medium had no detectable inhibitory effect on the growth of *Citrobacter in vitro*, which strongly indicates that hyaluronan inhibits the colonization of *C. rodentium* via indirect pathways through host or gut microbiota composition and functionality, but not by directly killing the enteropathogen *C. rodentium*. Increased probiotics may enhance host protection against pathogens through mechanisms, such as inhibition of pathogen mucosal adherence and invasion, production of antimicrobial substance, stimulation of pathogen-specific immunity, and competition for limited nutrients. Our results also provide evidence to show hyaluronan-enriched commensal *A. muciniphila* correlated with enhanced production of bacterial metabolites (SCFAs). Recent evidence indicated that SCFA treatment enhanced intestinal immune cell IL-22 production, contributing to the protection against *Citrobacter* infection and intestinal homeostasis.^38^ However, how precisely hyaluronan-induced microbiota metabolites (SCFAs) modulate host protection against enteropathogens warrants future investigation.

In conclusion, the results from our study demonstrated that orally administrated hyaluronan induces significant alterations in host gut microbiome composition, that the hyaluronan-induced microbiota can effectively confer protection against *Citrobacter* infection, and that *A. muciniphila* acts as an important mediator of this effect. These findings provide novel insights into the regulatory role of hyaluronan in modulating the gut microbiota composition and functionality and mucosal immunity in enteric infection and inflammation. Better understanding of the role of hyaluronan in the regulation of intestinal mucosal barrier properties will facilitate the development of novel therapeutics for many critical diseases that have broad and important public health implications.

## MATERIALS AND METHODS

### Preparation of hyaluronan

Hyaluronan (34 kDa, generously provided by Bloomage Biotechnology Corporation Limited, China) was dissolved at 4 mg/ml in the phosphate buffered saline (PBS), sterilized through a 0.2 micron filter, and kept at 4°C until use.

### Mice

Female BALB/c mice (six-week-old) were purchased from The Jackson Laboratory (Bar Harbor, ME, USA) and were fed autoclaved food and water, and housed in a controlled room (temperature, 68–73°F; relative humidity, 30–70%; lighting cycle, 12/12 h light/dark cycle) at Massachusetts General Hospital during the experimentation. All the experimental procedures were approved by the Institutional Animal Care and Use Committee of Massachusetts General Hospital, in accordance with guidelines issued by the recommendations in the Guide for the Care and Use of Laboratory Animals of the National Institutes of Health.

### C. rodentium *infection and the induction of bacterial colitis*

Mice were orally inoculated with *C. rodentium* (strain DBS100; American Type Culture Collection) to induce colitis as described in our published protocol.^39^ Briefly, bacteria were grown overnight in Luria broth (LB) and resuspended in PBS before infecting the mice (150 ul/mouse; 5 × 10^8^ CFU of *C. rodentium*). In order to evaluate the clearance of *C. rodentium*, we collected fecal pellets from each mouse every two days. The fecal pellets were weighed, serially diluted, and plated onto selective MacConkey agar. After overnight incubation at 37°C, bacterial colonies were counted.

### *Measurement of bacterial load in colon of* C. rodentium*-infected mice*

To assess the colonization of *C. rodentium* on the colonic epithelial surface, colon was collected after sacrificing mice, weighed, homogenized, and serially diluted and plated onto MacConkey agar plates. After 24 h, *C. rodentium* colonies were counted.

### Hyaluronan treatment

Following an initial acclimatation period, mice were randomly divided into four groups: Control group, Hyaluronan group (HA group), *C. rodentium*-infected group (CB group) and HA-treated and *Citrobacter*-infected group (HA+CB group). For the HA and HA+CB groups, all mice were given hyaluronan (600 μg/mouse,150ul/mouse) via oral gavage once a day during the experimental period, and the mice in the Control and CB group were treated with the same volume of PBS. After two weeks of intervention, each mouse from the CB group and HA+CB group was infected with *C. rodentium* (150 µl/mouse; 5 × 10^8^ CFU), and others left uninfected as controls. All mice were sacrificed 15 days post *C. rodentium* infection.

### Fecal microbiota transplantation

Fecal microbiota transplantation was performed based on our established protocol.^39^ Briefly, donor mice were randomly divided into two groups: PBS-fed group and HA-fed group. Mice in the HA-fed group received hyaluronan (600 μg/mouse, 150 µl/mouse) via oral gavage once a day, and mice from the PBS-fed group were treated at the same volume of PBS. After two-week treatment, fecal material (stool pellets, and cecal and colonic contents) from each mouse were collected 5 times over the course of two weeks under a laminar flow hood in sterile condition, and were mixed with PBS (200 mg/ml) and allowed to settle for 5 min before transfer to recipients. To remove soluble factors (e.g. hyaluronan) from the transferred material, fecal mixture was centrifuged (at 6000 rpm, 5 min). The centrifuged pellets were resuspended in PBS and then transferred to recipients. Recipient mice were randomly divided into two groups: Cont-F group (received microbiota from PBS-fed control donors), HA-F group (received microbiota from HA-fed donors). All the recipient mice were pre-treated with a cocktail of antibiotics (Kanamycin 0.4 mg/ml, Gentamicin 0.035 mg/mL, Colistin 850 U/mL, Metronidazole 0.215 mg/mL, Vancomycin 0.045 mg/ml) (Sigma Aldrich, St. Louis, MO) in drinking water for one week, a treatment regime that has been shown to effectively eliminate intestinal bacteria.^39,40^ Seven days after antibiotic treatment, each mouse was gavaged five times with a fecal slurry of stool pellets, and cecal contents from the PBS- and HA-treated donor mice. One week after the last microbiota transfer, some of the recipient mice from Cont-F and HA-F groups were infected with *C. rodentium* (150 ul/mouse; 5 × 10^8^ CFU) (Cont-F+ CB, HA-F+ CB), and others left uninfected as controls. The mice were sacrificed 12 days post *C. rodentium* infection.

### *Mice intervention study with* Akkermansia muciniphila

Culturing and preparation of *A. muciniphila* (ATCC® BAA-835™) were performed according to a standard propagation procedure (ATCC, Maryland, USA). Briefly, *A. muciniphila* was cultured in a brain heart infusion medium (ATCC® Medium 44) at 37°under strictly anaerobic conditions (100% N2). The cultures were centrifuged (8000 rpm, 5 min, 4°C), washed twice with sterile anaerobic PBS (pH = 7.4), and resuspended in anaerobic PBS (150 ul/mouse; 1 × 10^8^ CFU of *A. muciniphila*) by measuring absorbance at 600 nm half an hour before gavage. Notably, we cultivated *A. muciniphila* every day instead of culturing and freezing in advance, so as to ensure the vitality and function of bacteria. Then, Balb/c mice were pre-treated with a cocktail of antibiotics as described above, and gavaged with 1 × 10^8^ CFU *A. muciniphila* suspended in 150 ul or PBS per day throughout the experiment. After one week of treatment, all mice were infected by oral gavage with *C. rodentium* (150 ul/mouse; 5 × 108 CFU), and sacrificed 12 days post *C. rodentium* infection.

### DSS-induced colitis and oral administration of hyaluronan

Balb/c mice were orally gavaged with hyaluronan or PBS once a day for two weeks, and some of the mice were given drinking water containing 2.5% (w/v) DSS (molecular weight: 36,000–50,000 Da; MP Biomedicals, California, USA) ad libitum for 7 days, followed by distilled water for 3 days, and others left untreated as controls. Clinical parameters including body weight, bleeding, and stool consistency were recorded daily to calculate the disease activity index.^41^

### *In vitro bacterial cultures of* C. rodentium *with hyaluronan treatment*

*C. rodentium* were grown overnight in Luria broth (LB), resuspended in LB or LB containing different concentrations of hyaluronan (4 mg/mL and 0.4 mg/mL, respectively), and cultured at 37°C. Bacterial density was tested by measuring absorbance at 600 nm. Then, the growth curve of *C. rodentium* with or without hyaluronan treatment was obtained.

### *Fecal batch-culture fermentation* in vitro

Fecal batch-culture fermentation was performed based on previously published study.^42^ Briefly, fresh fecal samples from control mice were collected, weighed, and diluted in sterile PBS to prepare 20% (w/v) fecal slurry. The total volume of culture system was 5 ml. Hyaluronan treatment cultures consisted of 3.5 ml of culture medium (brain heart infusion medium), 0.5 ml of fecal slurry, and 1 ml of hyaluronan (dissolved in brain heart infusion medium, 4 mg/mL). Negative control cultures consisted of 4.5 ml of culture medium and 0.5 ml of fecal slurry. Batch cultures were incubated at 37°C anaerobically in a growth chamber (100% N2) without stirring, and samples were dynamically collected at 72 hours for DNA extraction and qPCR analysis of Firmicutes, Bacteroidetes, and *A. muciniphila*.

### In vitro *bacterial cultures of* A. muciniphila *with hyaluronan treatment*

*A. muciniphila* was cultured in a brain heart infusion medium at 37°under strictly anaerobic conditions (100% N2), resuspended in brain heart infusion or brain heart infusion containing different concentrations of hyaluronan (4 mg/mL and 0.4 mg/mL, respectively), and incubated at 37°C anaerobically. The level of *A. muciniphila* was obtained by measuring bacterial density at 600 nm at 72 hours.

### Histopathological examination

Colonic tissues were fixed in 4% buffered formalin, embedded in paraffin, and sectioned to 5 μm thickness. Hematoxylin and eosin (HE) staining was conducted according to standard methods, and colonic pathology was scored according to a modified histology scoring system based on our previously published methods.^43^ Goblet cells were stained with periodic acid-schiff (PAS) or AB/PAS following manufacturer instructions, and the number of goblet cells per crypt in the colon were quantified.

### Measurement of serum antibody

Mice were sacrificed 12 to 15 days after *C. rodentium* infection. Serum samples were collected, and the immunoglobulin G2a (IgG2a) level were measured as previously described.^44^

### Lymphocyte isolation and measurement of cytokine production

Mesenteric lymph nodes (MLNs) from all mice were harvested and placed in complete Dulbecco’s modified Eagle’s medium (cDMEM). Single lymphocyte suspensions were prepared from the MLNs by pressing the cells through a 70-μM nylon cell strainer (Falcon; BD Labware). Then, cells (5 × 10^6^ cells/ml) were cultured in 24-well plates in the presence of plate bound anti-CD3 monoclonal antibody (MAb; 10 μg/ml), and culture supernatants were assayed for Th1 (IFN-γ), Treg (IL-10), and Th17 (IL-17) cytokines as previously described.^39^

### Quantitative detection of colonic cytokine expression

Quantitative reverse transcription-PCR (qRT-PCR) was implemented as our previously published methods.^44^ Briefly, total RNA was isolated from colon tissue using TRIzol reagent (Invitrogen Life Technologies) according to the manufacturer’s recommendations, and reverse transcribed into cDNA using a PTC-200 Peltier Thermal Cycler (MJ RESEARCH). The expression of IL-1β, IL-10, IL-17, IL-22, IFN-γ, TNF-α, Muc1, Muc5, Muc13, Reg3γ, and Cramp were tested. GAPDH was the housekeeping control. The gene expression levels were calculated using the comparative 2^−ΔΔCt^ method. The primer sequences are listed in Table 1.

**Table 1.** Primers used in this study

Genes	Orientation	Sequence (5ʹ–3ʹ)
GAPDH	F	TGGAATCCTGTGGCATCCATGAAAC
	R	TAAAACGCAGCTCAGTAACAGTCCG
IL-1β	F	GATCCACACTCTCCAGCTGCA
	R	CAACCAACAAGTGATATTCTCCATG
IL-10	F	CCACAAAGCAGCCTTGCA
	R	AGTAAGAGCAGGCAGCATAGCA
IL-17	F	CCACGTCACCCTGGACTCTC
	R	CTCCGCATTGACACAGCG
IL-22	F	CAATCTTCCAGCAGCCATACA
	R	TCCTTAGCACTGACTCCTCG
IFN-γ	F	TCAAGTGGCATAGATGTGGAAGAA
	R	TGGCTCTGCAGGATTTTCATG
TNF-α	F	CCCTCACACTCAGATCATCTTCT
	R	GCTACGACGTGGGCTACAG
Muc1	F R	TCGTCTATTTCCTTGCCCTG ATTACCTGCCGAAACCTCCT
Muc5	F	AGAATATCTTTCAGGACCCCTGCT
	R	CACCAGTGCTGAGCATACTTTT
Muc13	F	AGAAACATTC CATGGCCTATCAA
	R	TGTCCA TAAACAGATGTGCCAAA
Reg3γ	F	TTCCTGTCCTCCATGATCAAAA
	R	CATCCACCTCTGTTGGGTTCA
Cramp	F	GCCGCTGATTCTTTTGACAT
	R	GCCAAGGCAGGCCTACTACT
*Universal*	F	TCCTACGGGAGGCAGCAGT
	R	GGACTACCAGGGTATCTAATCCTGTT
Firmicutes	Fc	GGAGCATGTGGTTTAATTCGAAGCA
	Ft	GGAGTATGTGGTTTAATTCGAAGCA
	R	AGCTGACGACAACCATGCAC
Bacteroidetes	Fa	GGAACATGTGGTTTAATTCGATGAT
	Fg	GGAGCATGTGGTTTAATTCGATGAT
	R	AGCTGACGACAACCATGCAG
*Akkermansia muciniphila*	F	CAGCACGTGAAGGTGGGGAC
	R	CCTTGCGGTTGGCTTCAGAT

### qPCR analysis of gut microbiota

Fecal material of mice were collected and DNA was extracted using the QIAamp DNA stool kit (QIAGEN, Chatsworth, CA). The abundance of bacteria was determined by qPCR using previously published methods,^36^ and the primer sequences are as Table 1.

### Gut microbiome analysis

Fresh fecal pellets were collected, and genomic DNA was extracted using the QIAamp DNA stool kit (QIAGEN, Chatsworth, CA) based on the manufacturer’s recommendations. Bacterial 16S rRNA genes were amplified at thermocycler PCR system (GeneAmp 9700, Applied Biosystems, Carlsbad, CA, USA) with the primers 338 F (5ʹ- ACTCCTACGGGAGGCAGCAG-3ʹ) and 806 R (5ʹ- GGACTACHVGGGTWTCTAAT-3ʹ). Purified V3-V4 amplicons library were pooled in equimolar and paired-end sequenced (2 × 300) on an Illumina MiSeq platform (Illumina, San Diego, USA) according to the standard protocols. Sequences with ≥97% similarity were assigned to the same Operational taxonomy units (OTUs) by Usearch (version 7.0). The community diversity (Simpson) was analyzed using Mothur (version v.1.30.1). β-Diversity was calculated based on the principal coordinate analysis (PCoA) to identify microbial differences among samples. A Venn diagram was implemented to show unique and shared OTUs. The microbial distribution was visualized using R package (version 2.15.3) based on community composition information at taxonomic levels. The dominant bacterial community difference between groups was detected using Wilcoxon rank-sum test or Kruskal–Wallis H test. The linear discriminant analysis (LDA) effect size (LEfSe) was performed online (http://huttenhower.sph.harvard.edu/galaxy). The Pearson analysis was used to measure the correlation between gut microbiota and metabolites, and the potential functional genes profiles of microbial communities in all samples were predicted with PICRUSt using STAMP (version 2.1.3) based on the Kyoto Encyclopedia of Genes and Genomes (KEGG) pathway database.

### Measurement of fecal short-chain fatty acids (SCFAs)

The levels of SCFAs in feces were measured by gas chromatography (GC) as previously reported.^45^ Briefly, 100.0 mg feces stored at −80°C were diluted with 1 mL vortex-mixed in Milli-Q water for 2 min, incubated at room temperature for 10 min, and centrifuged at 14,000 rpm for 20 min. The impurities in supernatants were removed by centrifugating twice at 14,000 rpm for 20 min, and 2-ethylbutyric acid (Sigma, MO, USA) was added as an internal standard. Then, the supernatants were transferred to into a fresh 2 ml glass vial for GC analysis. GC analysis was performed using an Agilent 7890 N gas chromatograph system (Agilent Technologies Inc., CA, USA) coupled with DB-FFAP capillary column (30 m × 0.25 mm×0.25 µm; Agilent). A flame ionization detector was used for identification and quantification of SCFAs in each sample. The data were acquired using the ChemStation software (Agilent). The concentration (in mM range) of SCFAs (acetic acid, propionic acid, isobutyric acid, butyrate acid, isovaleric acid, valeric acid, and hexanoic acid) were calculated using the linear regression equations (R2 ≥ 0.99) from the corresponding standard curves.

### Statistical analysis

All data are expressed as mean ± standard error of the mean (SEM). Statistical differences between two groups were determined using a two-tailed Student’s t-test. Datasets that involved more than two groups were assessed by one-way analysis of variance (ANOVA) test followed by Tukey’s multiple comparison’s test. Statistical analyses were performed using GraphPad Prism 8.01 (GraphPad Software, Inc., San Diego, CA, USA). Results with *P* < .05 were considered statistically significant.

## Data Availability

The raw sequencing data of this study had been deposited in the National Center of Biotechnology Information (NCBI) Sequence Read Archive (SRA) database under the BioProject accession number PRJNA717677. Additional data related to this paper may be requested from the authors.

## Teaser

Hyaluronan-induced microbiota confers protection against bacterial enteropathogen infection and intestinal inflammation
